# Trust and shared decision‐making among individuals with multiple myeloma: A qualitative study

**DOI:** 10.1002/cam4.4322

**Published:** 2021-10-05

**Authors:** Robin L. Whitney, Anne Elizabeth Clark White, Aaron S. Rosenberg, Richard L. Kravitz, Katherine K. Kim

**Affiliations:** ^1^ The Valley Foundation School of Nursing San Jose State University San Jose California USA; ^2^ Department of Family Medicine University of California San Diego La Jallo California USA; ^3^ UC Davis Comprehensive Cancer Center Sacramento California USA; ^4^ Department of Internal Medicine University of California, Davis Davis California USA; ^5^ Center for Health Policy and Research University of California, Davis Davis California USA; ^6^ Department of Public Health Sciences School of Medicine, University of California Davis California USA

**Keywords:** multiple myeloma, professional–patient relations, shared decision‐making, trust

## Abstract

**Background:**

Multiple myeloma (MM) is an incurable cancer with complex treatment options. Trusting patient–clinician relationships are essential to promote effective shared decision‐making that aligns best clinical practices with patient values and preferences. This study sought to shed light on the development of trust between MM patients and clinicians.

**Methods:**

Nineteen individual semi‐structured interviews were conducted with MM patients within 2 years of initial diagnosis or relapse for this qualitative study. Interviews were recorded and transcripts were coded thematically.

**Results:**

We identified three main themes: (1) *externally validated trust* describes patients’ predisposition to trust or distrust clinicians based on factors outside of patient–clinician interactions; (2) *internally validated trust* describes how patients develop trust based on interactions with specific clinicians. Internally validated trust is driven primarily by clinician communication practices that demonstrate competence, responsiveness, listening, honesty, and empathy; and (3) *trust in relation to shared decision*‐*making* describes how patients relate the feeling of trust, or lack thereof, to the process of shared decision‐making.

**Conclusion:**

Many factors contribute to the development of trust between MM patients and clinicians. While some are outside of clinicians’ control, others derive from clinician behaviors and interpersonal communication skills. These findings suggest the possibility that trust can be enhanced through communication training or shared decision‐making tools that emphasize relational communication. Given the important role trust plays in shared decision‐making, clinicians working with MM patients should prioritize establishing positive, trusting relationships.

## Abstract 2

Patient–clinician trust is influenced by both external factors and clinician behaviors in the context of multiple myeloma. Trust between patients with myeloma and their clinicians is an important aspect of shared decision‐making.

## 1 BACKGROUND

Multiple myeloma (MM) is an uncommon hematologic malignancy that primarily affects older adults, with around 35,000 new cases diagnosed annually in the United States.^1^, ^2^ Considered incurable, MM follows a relapsing and remitting course, where each period of remission is expected to become progressively shorter until treatment eventually fails.^3^ Management of MM involves a series of complex treatment decisions across the disease trajectory, with the goal of both prolonging life and maximizing quality of life.^4^ Many novel and effective therapies have been introduced in recent years, yielding multiple treatment options to address each relapse. However, responses to and side effects from treatments vary greatly, and few clear guidelines exist on which to base treatment decisions beyond first‐line therapy.^3^, ^5^ Further complicating treatment decisions, many therapies can be burdensome to patients in terms of their physical effects, financial impact, and frequency of administration or monitoring requirements.^5^, ^6^ Consequently, both clinical acumen and clear insight into patients’ goals, values, and preferences are needed to choose wisely.

Shared decision‐making—a process in which patients and clinicians partner to make treatment choices that optimize clinical outcomes while honoring individual values and preferences—is essential in circumstances like these, where there is limited or conflicting evidence to support which treatment is best.^7^, ^8^ While there is growing consensus that shared decision‐making is necessary to patient‐centered care for individuals with serious and life‐limiting illness,^9^ the best strategies to achieve shared decision‐making in practice remain elusive.^10^, ^11^ For effective shared decision‐making to occur, clinicians must be able to understand and effectively communicate the risks and benefits associated with various treatment options, while patients must feel empowered to communicate their goals and concerns.^12^ Trust between patients and clinicians is essential in this context.

Trust can be conceptualized both in terms of general trust in the medical profession or institutions as a whole, and as relating to specific interpersonal relationships between an individual clinician and patient.^13^ Importantly, interpersonal patient trust in clinicians has been found to influence decision‐making preferences and behaviors. In previous work among individuals with chronic conditions, lower level of trust in the clinician was independently associated with lower quality of shared decision‐making communication.^14^ Among hospital inpatients, trust in the clinician has been associated with patient‐reported shared decision‐making behavior.^15^ Similarly, among patients admitted to intensive care, trust in clinicians was correlated with dimensions of patient‐reported psychosocial care, including clinician shared decision‐making behaviors.^16^ In other work among individuals with breast cancer, however, trust in the oncologist was not associated with either patient‐reported or observer rated shared decision‐making.^17^ These disparate findings suggest that the relationship between trust and shared decision‐making is complex and challenging to quantify. Given that most studies of trust and shared decision‐making have been cross‐sectional in nature, the direction of the relationship between trust and shared decision‐making remains unclear. Does trust lead to better shared decision‐making, does shared decision‐making behavior promote trust, or is there a bi‐directional relationship? A recently developed model of relational trust in the clinician–patient relationship supports the idea that trust itself is dynamic. This model suggests that while an individual’s assumptions and predisposition to trust are the main factors leading to trust in a new clinician–patient relationship, interpersonal interactions over time reinforce or erode trust over time.^18^

In MM, specifically, greater trust in the healthcare system at large has been associated with more active communication patterns (e.g., asking clarifying questions, stating individual preferences) in healthcare encounters.^19^ Development of trust between clinicians and individuals with MM may be especially complex, given that they often receive care from a multidisciplinary team of clinicians, including medical oncologists, bone marrow transplant specialists, primary care providers, palliative medicine specialists, and advanced practice providers. In the context of life‐threatening illness, prior work suggests that trust in clinicians may develop in part as a coping response to vulnerability and distress.^20^ Further supporting this idea, a review on trust between cancer patients and clinicians found that patient trust in oncologists was generally high.^21^ Patient factors associated with higher trust across multiple studies included older age, female sex, and White, non‐Hispanic race/ethnicity.^21^ In more recent qualitative work, individuals with breast and prostate cancers described oncologists’ empathetic communication, thoroughness, and knowledgeability as enhancing trust.^22^ However, an ethnographic study among women with cancer found that trust was a dynamic across the trajectory of illness.^23^ While clinician communication did influence trust, the relational aspect of trust tended not to play a role until later in treatment. In contrast, on initial diagnosis trust was more “calculative,” derived from an individual’s assessment of websites, family and friend recommendations, availability or accessibility of the clinician, and urgency of commencing treatment. Relatively little is known about how individuals with MM, specifically, develop trust with clinicians.

The purpose of this study is to explore how individuals with MM describe the development of trust in clinicians and how they view trust as shaping treatment decisions or experiences. Results of this work can illuminate ways in which effective trust is established between patients and clinicians in the context of MM. These insights may help to inform future clinical and research efforts to strengthen trust and improve shared decision‐making among individuals with MM.

## 2 METHODS

This qualitative study was part of a larger project exploring patient and clinician experiences with shared decision‐making in MM. Individuals with MM and clinicians who care for individuals with MM were interviewed. In this paper, we report only findings from individuals with MM. Because of the rarity of MM, we used convenience sampling to recruit individuals who had been newly diagnosed or relapsed within the past 2 years. We recruited participants from both large, academic medical centers and smaller community‐based centers primarily in Northern and Central California, working with oncology staff to distribute pamphlets to eligible individuals, posting on multiple medical centers’ study pages, and disseminating recruitment flyers at MM support groups. Individuals received a $40 Amazon gift card in appreciation for their participation. Individual 1‐h semi‐structured phone interviews were conducted between October 2018 and February 2019. Two interviews were conducted in‐person at the patients’ requests. All interviews were recorded and transcribed verbatim. The UC Davis Medical Center Institutional Review Board approved this study.

This interview‐based study adhered to accepted criteria for reporting qualitative research (COREQ; Appendix A).^24^ The interview guide consisted of 11 open‐ended questions and was refined during data collection with suggested prompts (Appendix B). Before the interviews began, patients were told, “The purpose of this study is to understand the decision‐making processes for treatment of multiple myeloma from both the patient and clinician perspectives,” and that they had been chosen to participate due to their recent diagnosis or relapse with MM. Patients were asked to describe their experience being diagnosed and making decisions for initial and (if applicable) subsequent treatments. We asked about how treatment choices were presented and discussed, what factors were most important in making treatment decisions, and what role was played by the oncologist and family or friends during decision‐making. Patients were not presented with anything specifically about trust and/or shared decision‐making unless to probe further into a comment made by the participant. The decision to focus our analysis on trust and shared decision‐making was an inductively driven decision decided upon after all the interviews were conducted, and this analysis pertains only to portions of the interviews related to those topics.

The initial coding scheme was developed inductively.^25^ Two researchers (R.L.W. and A.E.C.W.) initially independently reviewed two randomly chosen transcripts and documented emerging themes. They then met to discuss, compare, and develop these emerging themes. They repeated this process three times, further developing their coding schema in Nvivo with parent and child subthemes. The coding scheme and any differences of opinion were reviewed with the PI. Upon consensus, they went back and independently recoded these initial six transcripts with the more finalized codebook and met again to compare codes. They then tested their codebook on an additional four transcripts to compare their coding and concluded that they were reaching consensus. The remaining nine interviews were coded independently by R.L.W or A.E.C.W. We noted that after 12 patient interviews we were seeing many similarities in experience and considered that we may be reaching theoretical saturation.^26^ Any disagreement in the application of the coding scheme was negotiated and a conclusion was achieved by consensus. The researchers and the PI met frequently to discuss the coding process. The three themes discussed in this manuscript are a subset of the themes inductively found in the data. These themes were selected by the entire research team as the focus for this manuscript.

## 3 RESULTS

We interviewed a total of 19 individuals with MM. Most of our sample identified as non‐Hispanic White (78.9%), between the ages of 55–64 (36.8%) or 65–74 (31.6%), and married (57.9%). Our participants were generally well‐educated, with 42.1% reporting a 2‐ or 4‐year college degree and 26.3% reporting a post‐baccalaureate degree. Approximately two thirds of participants identified as newly diagnosed with MM (63.2%). The sample was evenly divided between men (*n* = 9) and women (*n* = 10). Details of participant characteristics can be found in Table 1.

**TABLE 1:** Sociodemographic and clinical characteristics of sample participants, *n* = 19

Characteristic	*N* (%)
Age at interview (in years)	
45–54	3 (15.8)
55–64	7 (36.8)
65–74	6 (31.6)
75+	3 (15.8)
Sex	
Male	9 (47.4)
Female	10 (52.6)
Race/ethnicity	
Asian	1 (5.3)
Hispanic/Latino	2 (10.5)
Other	1 (5.3)
White	15 (78.9)
Marital status	
Married	11 (57.9)
Not married	8 (42.1)
Highest level of education	
Less than high school	1 (5.3)
High school or equivalent	3 (15.8)
Some college	2 (10.5)
College degree (Associate or Bachelor's degree)	8 (42.1)
Post‐baccalaureate degree	5 (26.3)
Annual household income	
Less than $35,000	5 (26.3)
$35,000–$49,999	2 (10.5)
$50,000–$74,999	3 (15.8)
$75,000–$99,999	1 (5.3)
$100,000 or greater	5 (26.3)
Prefer not to answer	3 (15.8)
Disease status	
Newly diagnosed	12 (63.2)
Relapsed	7 (36.8)

### 3.1 Qualitative themes

We identified several themes from participants’ descriptions of establishing trust with clinicians. While most individuals described some degree of trust, there were different ways in which individuals chose to trust. We organized themes into three broad categories—*externally validated trust*, *internally validated trust*, and *trust in relation to shared decision*‐*making*—and identified subthemes within each category.

*Externally validated trust* describes patients’ predisposition to trust or distrust their clinicians. *Externally validated trust* may reflect an overarching belief in the trustworthiness of medicine, or beliefs about the trustworthiness of specific institutions or clinicians, but relies on external factors or characteristics rather on interactions between patient and clinician. *Internally validated trust*, in contrast, describes how individuals develop trust in a specific clinician. *Internally validated trust* develops through interpersonal interactions between patients and clinicians when patients observe behaviors that validate trustworthiness. Trust in relation to *shared decision*‐*making* describes how patients relate feelings of trust, or lack thereof, to the process of decision‐making. Each theme is explained below with illustrative quotes. See Appendix C, Tables C1–C3, where additional supporting quotes are presented for each theme. Participant quotes are lightly edited for clarity (e.g., removing “um,” “er”).

#### 3.1.1 Externally validated trust

Within *externally validated*, we identified several subthemes: *trust in clinicians*, *trust in institutions*, and *personal beliefs or preferences*. Example quotes illustrating these themes are listed in Appendix Table C1.

##### Trust in clinicians

Several patients expressed a high level of trust in clinicians generally, particularly in MM specialists. These feelings of trust were often enhanced if trusted others (e.g., family, friends, or other patients) had endorsed the particular clinician as having a good reputation:

I started researching what multiple myeloma was. And I remember reading a statement, I don’t remember by whom, but saying that it’s very important to find a doctor who specializes in multiple myeloma. So, what I did was one of the times that the group was in [referring to the assigned group of clinicians on the inpatient oncology rotation]…And I said, Do you have a doctor on staff that specializes in MM? And they mentioned [my current doctor] And that’s how I started with [my doctor] and I like him very much… I believe in my doctor. I heard such good things about him from other people including the infusion nurses. (Pt02)

Patients whose initial diagnoses were precipitated by emergent or debilitating symptoms sometimes described their relationship with the clinicians as trusting because they simply had no other choice:

But back then [when I was first diagnosed] I was really scared. I didn’t know what to believe. I just kind of trusted them [the clinicians] because I didn’t have another choice. (Pt01)

One or two patients expressed a general sense of wariness about clinicians and their motivations:

I want for patients to be aware that there are doctors who don’t really care about you. They just want to make money (Pt16)

##### Trust in institutions

Many patients described particular institutions and clinicians within them as trustworthy, not because of first‐hand experiences, but because of their reputation or prestige. Often, affiliation with an academic center or particular specialization in MM inspired trust. One patient described why he was excited to change from his small‐town practice to a large, academic cancer center, because of access to clinical trials and new treatments, like chimeric antigen receptor (CAR) T‐cell therapy:

You know when you get passed on from a small town or an outlying city somewhere, right, and you get passed onto something like this [large academic medical center], like you just hit the major league. You know? (Pt08)

Conversely, a few individuals had pre‐existing perceptions of healthcare in general that made them hesitant to trust clinicians:

Everything I read about the medical system it just doesn’t inspire confidence, and it’s like, what’s the third leading cause of death in the US, you know? It’s people seeking out medical care. (Pt19)

##### Personal beliefs or preferences

Due to personal beliefs or preferences, patients also sometimes identified external characteristics that they associated with trustworthiness. For example, a female patient preferred finding a female physician because of her belief that “female doctors are more caring and more receptive to women’s opinions.” (Pt16) Preferences that patients had could be related to stereotypes or biases. For example, one patient expressed difficulty connecting with his primary care provider due to his accent:

And he – his English is fine, you could understand him but he’s got, you know he’s got an accent and what have you. And I’m not saying that you know I can’t have a good conversation in English with him but sometimes I felt like I wasn’t getting my point across as well as um, as I thought as well as I wanted it to or as well as I felt he ought to – he should understand it. (Pt12)

Another was excited to learn that his oncologist was of Indian descent, because of his belief that they are more competent clinicians:

And so we get in the office and [my new clinician] walks in and he’s Indian. And not that I’m a racist sort of thing but he walked in I thought, yes! I get the smart [expletive]. I’m in there, I’m gonna be around for a while! Yeah! You know? (Pt08)

#### 3.1.2 Internally validated trust

Most patients relied to some degree on *internally validated trust*, finding signs to validate trustworthiness in the actions and behaviors of clinicians that patients personally observed. Subthemes related to *internally validated trust* were often related to communication skills, and included: *competence*, *responsiveness*, *taking time & listening*, *honesty*, and *empathy*. Example quotes illustrating these themes are listed in Appendix Table C2.

##### Competence

Patients who sought out their own information about diagnosis and treatment options often used this knowledge as a way to triangulate their trust in the oncologist. Patients were reassured in their oncologists’ recommendations when they reflected knowledge of current evidence and new developments in treatment. Some patients expected detailed demonstrations of knowledge. For example, one patient who had performed independent research about options expected not only to hear that her oncologist was familiar with these options, but also that a detailed rationale would be provided to justify treatment recommendations:

I want to hear their rationale, like I’m moderately knowledgeable about the different protocols and I know there’s different mechanisms on different fields, like 3 main classes of drugs. And so I want to hear their rational. I want to hear their explanation about, okay, why pick this? Why pick this regimen over that regimen? Like, what’s your rationale for that? And I would want them to explain their rationale to me. (Pt11)

##### Responsiveness

Many individuals described confidence that the care team would be there when needed as key to promoting trust. Often, this was about the reliability of the care team in following up with abnormal results, or returning patient phone calls in a timely manner:

You can call over there and I get a call back or get an email right away. That’s probably a big thing for me too. (Pt10)

Conversely, feeling that they were being discounted or forgotten eroded trust: “My primary care physician when he came back from vacation he never followed up with me and I was very angry about that. He never called me, he never emailed, nothin’. It was almost like, well, it just sucks to be you.” (Pt09).

##### Taking time and listening

Overwhelmingly, patients expressed that oncologists who were willing to spend time listening to concerns without distraction inspired trust. One patient, for example, appreciated his oncologist’s willingness to disengage from the computer and make the effort to make complex information understandable to him.

He set back from the computer and just looked at me and started talking to me. And started talking to me in layman’s terms just so I could understand. I don’t understand all that blood work and stuff so he explained it. He explained what was going on. And he explained it in detail and with compassion. And then he showed me in papers, which I liked that, he just handed me papers. Because I’m not real computer literate. So he handed me paperwork and we read over it together. And so I could see that he had done his homework basically. And by the time we had that conversation I was ready to trust him, I was ready to trust him, um‐hum. (Pt05)

In contrast, many patients who did not feel trust described experiences where they perceived the oncologist was not willing to spend the time to ask questions and provide explanations. One patient who had gone through several oncologists before finding one that he trusted described always feeling rushed:

It’s your life and you know my oncologist spends 15 minutes with me and he’s just a whole lineup all day of people who are in and out of his office… And honestly I think the best way to put this is that I just felt like I was a cog in a wheel. (Pt09)

##### Honesty

Honest and transparent communication were also important in establishing trust between patients and oncologists. One patient described his clinician’s direct explanation of the disease and its trajectory as helping to lay the foundation for a positive relationship:

He explained the whole thing about multiple myeloma. He explained where it was, where it’s going, what they do. He just flat out laid it on the line. From then on me and him had like, we had the greatest relationship. (Pt08)

On the other hand, trust was eroded when patients perceived that the communication was not transparent. A patient who was initially treated in the hospital by a team of oncologists and residents described her perception that nobody wanted to be the one to deliver honest information about prognosis or negative effects of treatment. This led her to do her own research rather than just accepting treatment recommendations:

And I don’t know if that’s with all cancer patients but they just didn’t know what to say. They would kind of just sugar coat when they’d come in and talk to me. So no one really educated me on what myeloma was. I kinda had to self‐educate myself. (Pt10)

##### Empathy

Patients described oncologists’ positive attitudes as helping them to cope with uncertainty and eventual treatment failure:

But at the end of the day he always told me not to worry at all because if this particular one doesn’t work there’s another one. And I’m a very good candidate to be on another med or clinical trial. He’s always really so positive with me. (Pt01)

Communicating warmth and empathy was also important in developing trust. One patient described a physician’s ability to communicate empathetically as key in his decision to be treated there:

I don’t think we had long conversations but I think it was clear that he was sympathetic. He was, maybe empathetic. I had a similar experience with [my first oncologist] where it was all clinical and very dry and straightforward. And I just got none of that from [the first oncologist]. [The new oncologist] was very warm and easy to understand. (Pt15)

Similarly, one or two patients who were less trusting of their oncologists described them as competent, but not caring, or suspected them of having ulterior financial motives for their treatment recommendations:

Because I’ve met doctors [who] were so educated, you know or you’ll think oh you want them because he’s educated, he has experience. But they’re not caring doctors. They just know their stuff but they don’t really mean to help patients you know they just mean to help themselves. (Pt16)

#### 3.1.3 Trust in relation to decision‐making: concurrence with professional recommendations

Often for individuals with MM, shared decision‐making is not as straightforward as selecting between treatment options. Rather, patients choose a clinician and trust them to curate informed recommendations based on their specialized knowledge and attention to their patients’ values. When patients trusted that their clinicians understood their needs or had previous positive experiences relying on treatment recommendations it instilled confidence in treatment choices. However, patients who were more generally distrustful or who had prior negative experiences sometimes expressed a desire to conduct their own research or to switch care teams entirely. Example quotes illustrating this theme are listed in Appendix Table C3.

Many patients expressed confidence relying on decisions made by their clinicians when a trusting relationship had been established. One patient, for example, described her process deciding to participate in a clinical trial. She was eligible for several trials and also had the option not to participate, but ultimately deferred to her clinician’s recommendation because of her faith in him:

Really to be honest with you…I wasn’t weighing anything [in terms of treatment decisions]. I was just grateful to get into this trial. I believe in my doctor...[he] wanted me in this one, I got in it. He was happy; I was happy. So that was just my attitude about that. (Pt02)

Sometimes patients who tended to defer to the clinician’s recommendations explained that they did not have the time or capacity to question the clinician’s judgment or engage in a more complex decision‐making process:

It was pretty obvious that I couldn’t negotiate the intellectual portfolio that was gonna be required of me [to make a treatment decision]. So, plus I was really getting kind of looped out by all the chemo. So, at a certain point I just had to say, you know I trust this guy, I have to listen to what he says, because I don’t think I can make this decision myself. (Pt15)

However, some individuals use their previous positive experiences with the clinician’s treatment recommendations to validate their decision to trust in subsequent treatment recommendations:

Yes, honestly [I listen to whatever treatment my doctor recommends], because I didn’t think I was gonna live and since they saved me like they did I trusted them so extensively and I still do. Yes, because they know exactly every inch of me, every single cell. They’ve taken cells out of my body now and put cells in. I trust them extensively. (Pt01)

In contrast, patients who expressed lower trust in their physician were more apt to seek second opinions and do their own research rather than relying on their physician’s recommendation. One patient described a lack of trust in his first oncologist, resulting from his perception that the oncologist was always rushed, and gave generic “cookie‐cutter” treatment recommendations. Ultimately, this lack of trust led him to refuse treatment and switch clinicians.

They had this protocol that they used for everyone. He did a bone marrow biopsy in my hip and said, yeah, he confirmed it was myeloma. This is the treatment we’re gonna put you on. And I just did not have a rapport with him. And at that point I really started thinking about getting out of [my cancer center]. [Describing the new cancer center after switching, and why he trusted their recommendations more] He talked to me a lot about multiple myeloma, what it was. One of the things he said that was particularly reassuring was that this is something that’s very manageable. (PT09)

Another patient, similarly, sought out second opinions and ultimately decided against her oncologist’s recommended treatment because of her suspicion that they would receive a “commission” and therefore had an incentive to prescribe unnecessary treatments:

It’s supposed to be a patient/doctor decision. It’s not just the doctor telling the patient you’re gonna be treated…patients have the right to say no and to do their own research and be a part of the decision making. But this doctor was so adamant at me going on treatment…Well why would you treat a patient when they don’t need it unless you needed the extra money? So that’s why I want people to be aware that some doctors are like that. (Pt16)

A few patients were reassured by doing their own research, even as they generally accepted treatment recommendations from clinicians.

[My clinician] hasn’t ever suggested anything that’s so outlandish that [I would question his treatment recommendation]. And believe me I do get on the internet and you know research some of this stuff. But I – the thing I don’t do is read the horror stories [about different treatments]. Because I think that just kind of puts another nail in the coffin so to speak, because then you do start to worry if you read – if you read the negative stuff. (Pt14)

Some patients who went with clinician recommendations for chemotherapy researched and pursued concurrent integrative treatments. They wanted to discuss these with their clinicians even though they were choices that were not offered within the oncology care setting.

[My doctor] basically said it’s my decision [if I want to use naturopathic treatments]. He said if it was anybody else I would say, no way, but you know he knows how strong I am and I how much research I’ve done on this and he said, well as long as we continue to monitor your blood results go for it. And so I did. (Pt17)

After the initial treatment phase, patients often sought out information about potential upcoming treatment options. In this phase of treatment, patients typically understand that they will experience a relapse sooner or later and begin gathering information before needing to make a treatment decision.

[Explaining that even though he primarily relies on his clinician’s recommendations, he still wants to research different options in advance because he knows he will eventually relapse] Yeah, there are two clinical trials that I asked about because I’m pretty much at the end of my protocols. There’s one or two things that they can do if my [current treatment] fails but really they’re looking at a clinical trial for me next. So in the course of running out of options, I saw a couple things that I brought to my hematologist’s attention and he said he’d look into them. (Pt15)

[Explaining about the treatment they are currently on] After this [treatment] some people go into remission…I mean, it’s not curable, but [some are in remission] I think for 20–30 years; some people die in 2–3…and it’s a crapshoot. So it’s been a whirlwind of talking to people and researching and watching videos and doctors. I’m going to a conference on multiple myeloma and I’m super excited about it because it’s going to be for patients and caregivers and it’s going to help me a lot with the, just information [about what might come next]. (Pt03)

## 4 DISCUSSION

Among patients with MM, trust between patient and clinicians evolves throughout the treatment trajectory, but precisely how trust impacts the shared decision‐making process remains uncertain. More often than not, patients in our study deferred to clinicians’ treatment recommendations. However, the patient’s trust in the clinician was key in allowing them to accept the proposed treatment plan. While some prior quantitative work has found that greater trust in clinicians is associated with shared decision‐making,^27^, ^28^ our findings suggested that the relationship between trust and shared decision‐making may be more complex.

Our findings about how trust develops in MM are largely consistent with findings from previous qualitative work among individuals with cancer.^21^, ^23^ In particular, our themes of *externally and internally validated trust* are similar to the concepts of “calculative” (based on reputation) and “relational” (based on interactions) trust previously described by Yeh.^23^ Calculative and relational trust are described as evolving in a linear process, with the role of calculative trust waning and the role of relational trust growing over time. We found this to be relevant for individuals with MM as well, because it takes time and repeated interactions to develop *internally validated trust*. However, in the context of MM, there is additional complexity because of the chronic nature of the disease and the involvement of multiple clinicians over time. For example, an individual with MM may go through the process of establishing trust with a medical oncologist, but later require a transplant specialist, a palliative care clinician, or an oncologist who participates in clinical trials. In addition, MM is more likely to be diagnosed either emergently or after a lengthy period of vague symptoms compared with other cancers,^29^ especially common cancers which can be diagnosed through routine screening. In emergent cases, patients may have limited time for even calculative assessments of trustworthiness, while patients who experience lengthy workups or delayed diagnoses may be less disposed to trust clinicians.

The individual factors we identified as driving both externally and internally validated trust, such as reputation, honesty, empathy, and competence were also consistent with findings from previous work.^20^, ^21^, ^23^ However, one important external factor mentioned by several participants was finding a clinician who specializes specifically in MM, due to the complex nature of the disease. Finding a specialist may be more challenging in MM compared with more common cancers, particularly for individuals who live in rural or medically underserved areas. Future work might explore further how access to highly specialized clinicians impacts the development of trust in MM and other relatively uncommon cancers. Several factors that we identified as relating to *externally validated trust* are beyond the immediate control of the healthcare team, such as the individual patient’s perceptions of the oncologist or healthcare center’s reputation, and their pre‐existing preferences. However, these findings suggest that efforts to elevate the reputation of the clinician or organization within the community, such as through engagement with community organizations, could help to promote trust.

At the same time, the themes we identified around *internally validated trust* suggest that there are steps clinicians can take to improve trusting relationships. Consistent with previous work, our findings suggest that building trust involves specific patient communication approaches, such as taking adequate time for appointments, answering questions, appearing informed and knowledgeable, actively listening, and expressing empathy.^19^, ^21^, ^30^ Relatively few studies have examined interventions to increase trust, though physician communication skills training, physician disclosure of financial incentives, and patient–physician concordance in care beliefs have been associated with significant increases in patient trust in randomized controlled trials.^31^ While no patients in our study reported that their clinicians disclosed financial incentives, a few did express concerns that financial motives could underlie treatment recommendations, suggesting that transparency around such incentives may help to promote trust.

Patients’ descriptions of trust and decision‐making in our study also support the conceptualization of shared decision‐making in MM as being both an “interactional” as well as “transactional” process. As described by Epstein and Street,^32^ a transactional model of shared decision‐making is one in which patients and clinicians exchange information, often using a quantitative approach (e.g., decision aids that emphasize average treatment risks and benefits), to come to an agreement on a treatment decision. In an interactional model, on the other hand, clinicians and patients engage relationally and affectively in a process of shared deliberation, achieving what Epstein and Street term “shared mind.”^32^ Shared mind occurs when clinician and patient are attuned in their relationship, such that “new ideas and perspectives emerge through the sharing of thoughts, feelings, perceptions, meanings, and intentions.”^32^ Whereas shared decision‐making from a transactional perspective is often explicit (i.e., the patient makes a choice from among discrete options after reviewing information), interactional shared decision‐making can be more implicit, in that decisional preferences are conveyed through attunement and “shared mind” rather than in a series of individual choices.

While many patients in our study described transactional decision‐making interactions, the concepts of interactional decision‐making and “shared mind” also resonate with our participants’ descriptions of feeling known or seen by their oncologists and the confidence such feelings inspired in the oncologists’ treatment recommendations. Thus, while it may appear that some patients simply defer to oncologists’ recommendations out of blind trust, it is possible that for others this deference follows a process of affective engagement and information‐sharing that has allowed the oncologist to present tailored information and treatment recommendations informed by an understanding of patient values. In the former scenario, trust may be viewed as a barrier to shared decision‐making because it leads the patient to accept treatment recommendations without question. In the latter scenario, trust is essential to achieving the attunement between clinicians and patients that is necessary for “shared mind” to occur.

Several previous studies have pointed out that trust could have a negative impact on shared decision‐making.^23^, ^33^, ^34^ Yeh suggest that as relational trust grows, some patients cease participation in the decision‐making process and give complete control to their clinicians.^23^ Helmes et al. found, similarly, that higher trust was associated with preferring to leave medical decisions to the clinician, raising the concern that trust may not actually lead to this preference (consistent with an interactional shared decision‐making model), but rather that patients might express trust as a way of justifying their lack of interest in participating actively in the decision‐making process.^33^ Alternatively, Kraetschmer et al.^34^ observe that there may be optimal levels of trust, such that both very high and very low levels of trust may work counter to shared decision‐making processes. They observed that individuals whose trust was high but not “blind” had decisional preferences most aligned with shared decision‐making principles, whereas individuals with “blind” trust preferred a very passive role and individuals with low trust preferred an autonomous role.^34^ We found, similarly, that our participants’ descriptions of trust in relation to shared decision‐making ranged from very trusting to very distrusting of treatment recommendations, with many individuals falling somewhere in between. Further supporting Kraetschmer et al.’s findings, the few individuals who were highly distrustful seemed not to engage in decision‐making discussions with clinicians they distrusted at all, preferring to seek out second opinions or switch care providers altogether.

The clinician–patient relationship in a disease with a long‐term treatment trajectory like MM, may lend itself particularly well to an interactional model of shared decision‐making, because of the opportunity to build trust and strong patient–provider relationships over time. The complexity of decision‐making in MM may nudge patients toward an interactional decision‐making approach; given multiple, rapidly changing treatment options, a purely transactional decision‐making approach may be too daunting for many patients. If this is the case, it may be that for many individuals with MM finding a clinician they trust is the most important choice, facilitating interactional decision‐making as the clinician–patient relationship develops across the trajectory of care. This interpretation is supported by our finding that many patients sought out information about future treatment options and wanted to discuss them with clinicians, even as they reported generally deferring to treatment recommendations when it was time to decide. Indeed, this anticipatory aspect of decision‐making may be a feature unique to MM and other incurable cancers, as the inevitability of eventual relapse leads patients to pre‐emptively seek information about future treatment options. While our study suggests that most individuals with MM are generally trusting of their clinicians and their treatment recommendations, the extent to which these feelings reflect interactional shared decision‐making as opposed to a “blind” trust and preference for less autonomous decision‐making are not entirely clear. While our study was not designed to examine changes in trust or shared decision‐making over time, studies of longitudinal clinician–patient relationships in chronic cancers could shed further light on the development of trust and its role in shared decision‐making in this context.

Our study has several limitations. Our qualitative interviews were designed to elicit rich data related to decision‐making in MM rather than to produce generalizable findings. Given that our sample largely identified as White, highly educated, and healthy enough to participate in this study, it is possible that trust and decision‐making operate differently in other populations and for other conditions. In fact, differences are likely given our finding that trust was influenced by personal beliefs and preferences, and evidence from prior work that culture, language, race, and ethnicity impact trust and shared decision‐making.^35^, ^36^ While our study sheds light on factors that influence trust and the relationship of trust to shared decision‐making, the extent to which patient–clinician trust and shared decision‐making can be improved is unclear. Future work might explore relationships of trust and shared decision‐making among more diverse groups of MM patients, and examine the effect of communication training or similar interventions on trust and shared decision‐making.

Despite these limitations, our study offers new insight into the ways in which trust develops and impacts shared decision‐making in the context of patient–clinician relationships in MM. While our findings suggest there are many possible approaches for building trust in clinician–patient relationships, establishing trust is an essential step on the path to effective shared decision‐making.

## ETHICS STATEMENT

This research was reviewed and approved by the Institutional Review Board (IRB) of the University of California, Davis.

## CONFLICT OF INTEREST

None of the study authors have conflict of interest to report.

## Data Availability

Research data are not available to be shared due to protect participant privacy.

## APPENDIX A

### COREQ 32‐ITEM CHECKLIST

**Table:**

Domain 1: Research team and reflexivity	
Personal characteristics	
1. Interviewer/facilitator	Two authors, Drs. Robin L. Whitney and Anne E. C. White conducted the in‐depth interviews.
2. Credentials	Dr. Whitney received her BA in English and German from Bowdoin College, her BS in Nursing from the University of Southern Maine, and her PhD in Nursing Science and Healthcare Leadership from UC Davis. Dr. White received her AB in Sociology from Princeton University and her MA and PhD in Sociology from UCLA.
3. Occupation	Dr. Whitney is an assistant professor at The Valley Foundation School of Nursing at San Jose State University. Dr. White was a postdoctoral researcher and fellow at the Center for Healthcare Policy and Research and in the Department of Internal Medicine at UC Davis Medical Center.
4. Gender	Drs. Whitney and White identify as female.
5. Experience and training	Dr. Whitney has had training and experience conducting qualitative interviews and data analysis as a graduate research assistant at UC Davis and has subsequently been a co‐investigator on several qualitative research projects involving patient experiences. Dr. White has conducted qualitative research for 15+ years. Her dissertation and post‐doctoral research have focused on medical sociology, doctor–patient interactions, and qualitative methodologies.
Relationship with participants	
6. Relationship established	Researchers had no prior relationship with participants.
7. Participant knowledge or researchers	Participants had no prior knowledge of the researchers.
8. Interviewer characteristics	The researchers’ methodological training is reported.
Domain 2: Study design	
Theoretical framework	
9. Methodological orientation and theory	The methodological orientation that underpins the study is thematic analysis.
Participant selection	
10. Sampling	This study used convenience sampling.
11. Method of approach	We recruited participants from both large, academic medical centers and smaller community‐based centers primarily in Northern and Central California, working with oncology staff to distribute pamphlets to eligible individuals, posting on multiple medical centers’ study pages, and disseminating recruitment flyers at multiply myeloma support groups.
12. Sample size	Nineteen participants.
13. Non‐participation	No participants refused to participate or dropped out of the study.
Setting	
14. Setting of data collection	Researchers collected the data via phone interviews for 17 participants. Two participants were interviewed in‐person (at their request) in a reserved office on campus at UC Davis Medical Center.
15. Presence of non‐participants	For the two in‐person interviews, no non‐participants were present. For the 17 interviews conducted via the phone, only one participant had his spouse actively co‐present (on speaker phone) during the interview, but understood that only his responses would be transcribed.
16. Description of sample	We interviewed a total of 19 individuals with MM. Most of our sample identified as non‐Hispanic White (78.9%), between the ages of 55–64 (36.8%) or 65–74 (31.6%), and married (57.9%). Our participants were generally well‐educated, with 42.1% reporting a 2‐ or 4‐year college degree and 26.3% reporting a post‐baccalaureate degree. Approximately two thirds of participants identified as newly diagnosed with MM (63.2%). The sample was evenly divided between men (*n* = 9) and women (*n* = 10).
Data collection	
17. Interview guide	Participants were not provided with an interview guide before the interview. The interview guide was pilot tested with members of the research team. The interview guide was reviewed and updated during data collection.
18. Repeat interviews	No repeat interviews were conducted.
19. Audio/visual recording	Audio recordings were collected.
20. Field notes	Researchers wrote brief summaries and highlights of interviews immediately following the interviews.
21. Duration	Interviews lasted for 1 h.
22. Data saturation	After we had interviewed seven patients and had received their corresponding transcripts, we began to review the interviews and conduct initial analysis. We assessed that after 12 patients we had reached data saturation. If additional patients then contacted us for interviews, we still interviewed them as we wanted to include as many individual experiences as possible to illustrate the broadest range.
23. Transcripts returned	Transcripts were not returned to participants.
Domain 3: Analysis and findings	
Data analysis	
24. Number of data coders	Two researchers, Drs. Whitney and White directly coded the data. They met bimonthly with the PI, Dr. Kathy Kim, and the project’s research assistant, Nilpa Shah, to discuss and refine themes. The whole research team (all co‐authors) met quarterly to discuss larger themes found.
25. Description of coding tree	Coding was conducted in Nvivo with larger “parent” themes and nested “child” themes.
26. Derivation of themes	Themes were inductively derived from the data.
27. Software	Nvivo
28. Participant checking	Participants did not provide feedback on the findings.
Reporting	
29. Quotations presented	Participants quotations are provided to illustrate the themes. Each quotation is identified with the participant ID number.
30. Data and findings consistent	There is consistency between the data presented and findings. Quotations were selected for their representation of the data.
31. Clarity of major themes	Our three major themes (and subthemes) are clearly presented in the findings along with definitions or explanations.
32. Clarity of minor themes	Yes, diverse cases are included in the findings. For example, in the “personal beliefs or preferences” subtheme, we provide examples how physicians’ ethnicities can serve both as a positive and as a negative attribute for their patients.

## APPENDIX B

### INTERVIEW GUIDE

**Table:**

**Question #1: Can you tell me a little about yourself? For example, anything you would like to share about your work, family, or the area you currently live in?**
**Question #2: Thinking back to before you were diagnosed with Multiple Myeloma, were you having any signs or symptoms? Can you describe what you were you experiencing, what made you see a doctor initially?** Possible Probes: How long were those symptoms occurring? What did you think was going on? Who did you bring these up with, if anyone?
**Question #3: Would you tell me about the process you went through as you were being diagnosed? What steps did you go through and with whom? (I’m asking right now about diagnosis. I’ll ask you about treatment options next)** Possible Probes: When were you diagnosed? What type of medical provider diagnosed you? What was your relationship with that provider (doctor you already knew, someone you were referred to, first time you met her?) Was a specialist involved, and how long did it take to see a specialist (i.e., referred)? Were family and/or friends involved with any of these steps?
**Question #4: Now, I’d like to talk about treatment options and plans. Can you describe how those discussions about treatment options went? What steps did you go through, what kind of information, and with whom?** Possible Probes: Where are you in your course of treatment? How many visits have you had with your oncologist? How often are you seeing them? Are there other doctors you are seeing related to your myeloma? Who was involved, doctors, nurses, other clinicians? What kind of information did you receive? Pamphlets, websites, verbal, etc? Were family and/or friends involved with any of these steps?
**Question #5: When you had questions or concerns as you were learning about your diagnosis and treatment options, how did you get those questions answered?** Possible Probes: Can you give me examples of specific issues that you remember? Who did you ask? Did you get what you needed?
**Question #6: Did you need any information beyond what your healthcare team gave you either about diagnosis or treatment? If so, what kinds of information or education did you yourself look for about the diagnosis or treatment?** Possible Probes: What kind of information were you looking for? Can you give me an example of something you looked for yourself? What sources of information did you use to educate yourself? (i.e., websites, library/journals, doctors, social media, support groups, and family members) Did your method of education change over time? What was the most useful information? Did any of the information you looked at change your decisions?
**Question #7: Did your family and friends participate in making your decisions about treatment? If so, can you tell me about what role they played?** Possible Probes: Who was involved and how? Did they attend visits, talk on the phone, find educational resources, or other activities? What did they discuss with you, your healthcare team, among themselves?
**Question #8: What were the important considerations in deciding which treatment you would undergo?** Possible Probes: What made you choose your option (were there trade‐offs that you had to consider)? Who do you think these considerations were important to, you, family, clinicians, etc.? What made you feel you could trust your clinician? Did you seek a second opinion? Why or why not?
**Question #9: What do you think are important factors for other patients to consider when making a treatment choice?** Possible Probe: Are there different considerations for different types of people? Having your current knowledge about the disease and treatment would you change anything?
**Question #10: What is your understanding of the current status of your multiple myeloma?** Possible Probes: How do you and your doctor communicate about your status? Do you and your doctor talk about monitoring and future expectations (if Y/ at what point did these discussions begin)? Is there anything that you’re tracking on your own (symptoms from tx, markers)? What sources of information did you use to help monitor or track your multiple myeloma and treatment symptoms? (i.e., websites, library/journals, doctors, social media, support groups, and family members) Did your method of monitoring/tracking change over time?
**Question #11: Is there anything else you would like to share that we haven’t already covered?**

## APPENDIX C

### DETAILED QUOTE TABLES

**TABLE C1:** Externally validated trust

	Example quotes
**Trust in clinicians** *In the initial stages of treatment, trust in the clinician may be based on reputation and advice from trusted others, may arise from an overarching belief in the trustworthiness (or lack thereof) of clinicians in general*.	**Trust Gained** *But back then [when I was first diagnosed] I was really scared. I didn’t know what to believe. I just kind of trusted them [the clinicians] because I didn’t have another choice*. (Pt01) *[explaining why he felt confident with his clinician and cancer center] Well my wife works in the medical field so she knows a lot of the doctors. Who’s good, who’s not and stuff*. (Pt08) *I started researching what multiple myeloma was. And I remember reading a statement, I don’t remember by whom, but saying that it’s very important to find a doctor who specializes in multiple myeloma. So, what I did was one of the times that the group was in [referring to the assigned group of clinicians on the inpatient oncology rotation]…And I said, Do you have a doctor on staff that specializes in MM? And they mentioned [my current doctor] And that’s how I started with [my doctor] and I like him very much… I believe in my doctor. I heard such good things about him from other people including the infusion nurses*. (Pt02) **Trust Eroded** *I want for patients to be aware that there are doctors who don’t really care about you. They just want to make money* (Pt16)
**Trust in institutions** *Reputation of the specific institution and attitude about healthcare institutions in general are important factors in determining trustworthiness*.	**Trust Gained** *You know when you get passed on from a small town or an outlying city somewhere*, *right*, *and you get passed onto something like this* [large academic medical center], *like you just hit the major league*. *You know?* (Pt08) *At a place as big as* [this academic cancer center] *they are consulting with the most highest and*, *qualified myeloma experts who deal with hundreds of myeloma patients in a year*. (Pt11) **Trust eroded** *Everything I read about the medical system it just doesn’t inspire confidence*, *and it’s like*, *what’s the third leading cause of death in the US*, *you know? It’s people seeking out medical care*. (Pt19)
**Personal beliefs or preferences** *Individuals have preconceived ideas about how trustworthy a clinician might be based on their own cultural or personal beliefs and preferences*.	**Trust gained** *And so we get in the office and* [my new clinician] *walks in and he’s Indian*. *And not that I’m a racist sort of thing but he walked in I thought*, *yes! I get the smart* [expletive]. *I’m in there*, *I’m gonna be around for a while! Yeah! You know?* (Pt08) *I’d rather see a female doctor*. *I think in general female doctors are more caring and more receptive to women’s opinions*. (Pt16) **Trust eroded** [Describing strained relationship with his primary care provider] *And he – his English is fine*, *you could understand him but he’s got*, *you know he’s got an accent and what have you*. *And I’m not saying that you know I can’t have a good conversation in English with him but sometimes I felt like I wasn’t getting my point across as well as um*, *as I thought as well as I wanted it to or as well as I felt he ought to – he should understand it*. (Pt12)

**TABLE C2:** Internally validated trust

	Example quotes
**Competence** *Signs of competence (up to date*, *articulating rationale) are sought to validate trustworthiness*.	**Trust Gained** *Like my doctor he’s so young but he’s very knowledgeable*. *He’s up*‐*to*‐*date on the most current treatments and plans*, *you know*, *and I just felt very comfortable with him and his whole team*. (Pt10) **Trust Eroded** *I want to hear their rationale*, *like I’m moderately knowledgeable about the different protocols and I know there’s different mechanisms on different fields*, *like 3 main classes of drugs*. *And so I want to hear their rational*. *I want to hear their explanation about*, *okay*, *why pick this? Why pick this regimen over that regimen? Like*, *what’s your rationale for that? And I would want them to explain their rationale to me*. (Pt11)
	
**Responsiveness** *Trust is validated through experiences of reliability and responsiveness from the clinician*/*care team*.	**Trust gained** *Yeah*, *once or twice a month I’ll either see* [my oncologist] *or …a fantastic nurse practitioner*. *She knows so much*. *So I feel very*, *I mean I like to see* [my oncologist] *but I feel very confident in seeing her if he can’t make it*. *So I’m always covered*. (Pt02) *You can call over there and I get a call back or get an email right away*. *That’s probably a big thing for me too*. (Pt10) **Trust eroded** *My primary care physician when he came back from vacation he never followed up with me and I was very angry about that*. *He never called me*, *he never emailed*, *nothin’*. *It was almost like*, *well*, *it just sucks to be you*. (Pt09)
	
**Taking time/Listening** *Trust was promoted when clinicians were present*, *engaged and willing to take time listening to the patient*.	**Trust gained** *He set back from the computer and just looked at me and started talking to me*. *And started talking to me in layman’s terms just so I could understand*. *I don’t understand all that blood work and stuff so he explained it*. *He explained what was going on*. *And he explained it in detail and with compassion*. *And then he showed me in papers*, *which I liked that*, *he just handed me papers*. *Because I’m not real computer literate*. *So he handed me paperwork and we read over it together*. *And so I could see that he had done his homework basically*. *And by the time we had that conversation I was ready to trust him*, *I was ready to trust him*, *um*‐*hum*. (Pt05) *Like my oncologist now she’s very detailed and she asks a lot of questions and she writes down every single thing that I say*. *I mean hand writing*, *not just on the computer*. *She’s the very first oncologist that I saw was writing all the time when I talked to her*. *She really*, *she really cares about how the progression*, *the way I feel*. *And it’s just the way the whole process is*. *Instead of rushing*, *instead of rushing like*, *okay*, *how do you feel?* (Pt16) **Trust eroded** *If I was a doctor and I diagnosed somebody with MGUS* [monoclonal gammopathy of undetermined significance, a benign condition that may progress to MM] *I would at least sit down with them and explain what this whole thing was*. *The doctor didn’t even do that*. *Because it’s not of interest to him and from his standpoint I’m of interest to him only if he was going to sell me drugs or send me for some extensive procedures or something like that*. (Pt19) *It’s your life and you know my oncologist spends 15 minutes with me and he’s just a whole lineup all day of people who are in and out of his office… And honestly I think the best way to put this is that I just felt like I was a cog in a wheel*. (Pt09) *Because now that I’ve been through*, *this is my fourth oncologist already who I’m seeing now*, *I see the difference*. *But with* [the first one] *it was just*, *okay*, *let’s watch*. *And he was always in a hurry to get out of the the visit*. (Pt16)
**Honesty** *Patients trusted clinicians who provided frank and honest information about what to expect*.	**Trust gained** *He explained the whole thing about multiple myeloma*. *He explained where it was*, *where it’s going*, *what they do*. *He just flat out laid it on the line*. *From then on me and him had like*, *we had the greatest relationship*. (Pt08) *You know and it’s the infusion I think the nurses in the Infusion Center are the ones that are the most honest because they see it*, *you know they see it one*‐*on*‐*one you know with the patients they deal with them*. *So that’s you know that’s kind of been a good resource for me too is my infusion nurse that I’ve had because she’s dealt with so many myeloma patients*. *And you know I didn’t understand that myeloma was just such a painful cancer and you know she’s like*, *oh*, *no*, *that’s normal you know and you know anytime I had a little ache and pain with it you know she was explaining me you know that this is normal and that’s what you’re – you’re gonna expect*. (Pt10) **Trust eroded** *And I don’t know if that’s with all cancer patients but they just didn’t know what to say*. *They would kind of just sugar coat when they’d come in and talk to me*. *So no one really educated me on what myeloma was*. *I kinda had to self*‐*educate myself*. (Pt10)
**Empathy** *Individuals trusted the intentions of their clinicians more when they felt that the clinician truly cared for them*. *Affective communication*.	**Trust gained** *But at the end of the day he always told me not to worry at all because if this particular one doesn’t work there’s another one*. *And I’m a very good candidate to be on another med or clinical trial*. *He’s always really so positive with me*. (Pt01) *And so the atmosphere was upbeat and very caring*. *They’ve learned your name*, *they knew what you were going through*. *And when you walked in they didn’t even flinch*. *I mean they never looked at you like*, *oh gosh*, *here comes that bald woman again*, *with all the curiosity that those people look at you with*. (Pt04) *I don’t think we had long conversations but I think it was clear that he was sympathetic*. *He was*, *maybe empathetic*. *I had a similar experience with* [my first oncologist] *where it was all clinical and very dry and straightforward*. *And I just got none of that from* [the first oncologist]. [The new oncologist] *was very warm and easy to understand*. (Pt15) **Trust eroded** *Because I’ve met doctors were so educated*, *you know or you’ll think oh you want them because he’s educated*, *he has experience*. *But they’re not caring doctors*. *They just know their stuff but they don’t really mean to help patients you know they just mean to help themselves*. (Pt16)

**TABLE C3:** Trust in relation to shared decision‐making

	Example quotes
**Trust promotes concurrence with physician recommendations** Very trusting individuals believe that that their clinicians understand their needs and have the expertise to make wise treatment decisions that are in their best interest, while very distrusting individuals are more skeptical of relying on clinicians’ treatment recommendations.	**Trusting in clinicians’ expert knowledge for treatment decisions** *Really to be honest with you…I wasn’t weighing anything* [in terms of treatment decisions]. *I was just grateful to get into this trial*. *I believe in my doctor*...[he] *wanted me in this one*, *I got in it*. *He was happy*; *I was happy*. *So that was just my attitude about that*. (Pt02) *It was pretty obvious that I couldn’t negotiate the intellectual portfolio that was gonna be required of me* [to make a treatment decision]. *So*, *plus I was really getting kind of looped out by all the chemo*. *So*, *at a certain point I just had to say*, *you know I trust this guy*, *I have to listen to what he says*, *because I don’t think I can make this decision myself*. (Pt15) *I tell you it’s amazing how doctors work and that they coordinate with other doctors. And they really do see your needs. And a good qualified doctor does make good decisions for you. And you know it’s good to be able to trust in those. I love that. I love the fact that I was able to trust like that*. (Pt05) *I was still in the situation of not having that much chance to research yet multiple myeloma*. *So I kind of just went by the doctor and his recommendations*. (Pt12) **Prior positive experiences can increase trust in treatment recommendations** *I just rely on my doctor’s reputation and the feeling that I have of comfort with her and her and uh*, *her expertise*. *And*, *consequently*, *when she has told us something that she feels has to be done*, *or we should do*, *or something we should think about*, *or the possibilities of how about if we try this or that*, *we’ve gone with it because so far she’s been right*. (Pt18) *Yes*, *honestly* [I listen to whatever treatment my doctor recommends], *because I didn’t think I was gonna live and since they saved me like they did I trusted them so extensively and I still do*. *Yes*, *because they know exactly every inch of me*, *every single cell*. *They’ve taken cells out of my body now and put cells in*. *I trust them extensively*. (Pt01) **Prior negative experiences can lead to greater questioning of treatment recommendations** *I always research everything*. *And especially more so now because we have gone through all this different … just the negative*, *uh experience with* [my previous clinician]. [This participant is referring to a treatment recommendation that was disputed in a second opinion by a clinician with specific expertise in MM]. *Now it’s almost like*, *oh my God I don’t trust you I’m going research it myself… I always listen to what they say but then I always do my own research*. (Pt16) **Some individuals with MM want to discuss their own research with the clinician, even if they tend to defer to the clinician’s recommendations** Examples with immediate treatment decisions‐independent research reassures the patient in accepting decisions [My clinician] *hasn’t ever suggested anything that’s so outlandish that* [I would question his treatment recommendation]. *And believe me I do get on the internet and you know research some of this stuff*. *But I –the thing I don’t do is read the horror stories* [about different treatments]. *Because I think that just kind of puts another nail in the coffin so to speak*, *because then you do start to worry if you read –if you read the negative stuff*. (Pt14) *I got lots of information* [before deciding to get a stem cell transplant]. *I got it from the* [medical center]. *I got it from going to my support groups because they have a setup there with all kinds of literature*, *pamphlets*, *booklets*. *And I read up on it and I certainly went online and looked at it*. *And*, *although stem cells aren’t for everyone*, *I thought I could handle it well enough*. (Pt13) *I keep notepads all over my house*. *And*, *I write down a question whenever I have anything*. *They* [clinicians] *just kind of give you the basics and uh*, *and they don’t get you a whole lot more* [referring to information about treatment options], *I don’t think*. *I mean they tell you the risk*, *and they always say*, *there’s no guarantee*. (Pt13) Examples with anticipatory decisions (knowing relapse will eventually happen) [Explaining that even though he primarily relies on his clinician’s recommendations, he still wants to research different options in advance because he knows he will eventually relapse] *Yeah*, *there are two clinical trials that I asked about because I’m pretty much at the end of my protocols*. *There’s one or two things that they can do if my [current treatment] fails but really they’re looking at a clinical trial for me next*. *So in the course of running out of options*, *I saw a couple things that I brought to my hematologist’s attention and he said he’d look into them*. (Pt15) [Explaining about the treatment they are currently on] *After this* [treatment] *some people go into remission…I mean*, *it’s not curable*, *but [some are in remission] I think for 20*–*30 years*; *some people die in 2*–*3…and it’s a crapshoot*. *So it’s been a whirlwind of talking to people and researching and watching videos and doctors*. *I’m going to a conference on multiple myeloma and I’m super excited about it because it’s going to be for patients and caregivers and it’s going to help me a lot with the*, *just information* [about what might come next]. (Pt03) *I’ve gotten more proactive and active in seeking information* [about treatments]. *For example*, *I’m going to go to a conference*, *a week from Saturday in San Francisco*, *and there are you know presenters who are going to be talking about the latest developments in myeloma treatment*. *They’ll be talking about the car T cell procedure*. *So*, *I am seeking out information more actively now than I have in the past and I’m getting better* (Pt09) Examples with seeking information about complementary/integrative treatments *[My doctor] basically said it’s my decision* [if I want to use naturopathic treatments]. *He said if it was anybody else I would say*, *no way*, *but you know he knows how strong I am and I how much research I’ve done on this and he said*, *well as long as we continue to monitor your blood results go for it*. *And so I did*. (Pt17) **Individuals who are less trusting of clinicians may not only do independent research, but also seek out second opinions and/or switch care teams**
	[explaining why she was skeptical of her first clinician’s treatment recommendation and sought a second opinion] *It’s supposed to be a patient*/*doctor decision*. *It’s not just the doctor telling the patient you’re gonna be treated…patients have the right to say no and to do their own research and be a part of the decision making*. *But this doctor was so adamant at me going on treatment…Well why would you treat a patient when they don’t need it unless you needed the extra money? So thats why I want people to be aware that some doctors are like that*. (Pt16) [explaining why he switched cancer centers] *They had this protocol that they used for everyone*. *He did a bone marrow biopsy in my hip and said*, *yeah*, *he confirmed it was myeloma*. *This is the treatment we’re gonna put you on*. *And I just did not have a rapport with him*. *And at that point I really started thinking about getting out of [my cancer center*]. [Describing the new cancer center after switching, and why he trusted their recommendations more] *He talked to me a lot about multiple myeloma*, *what it was*. *One of the things he said that was particularly reassuring was that this is something that’s very manageable*. (Pt09)
